# Efficacy of fasudil for the treatment of aneurysmal subarachnoid hemorrhage: A systematic review protocol of randomized controlled trial

**DOI:** 10.1097/MD.0000000000016885

**Published:** 2019-08-30

**Authors:** Hao-yan Wang, Guang-fu Song, Hong-wei Yang, Xue-feng Chang, Ren-bo Shen, Fu-yi Yang

**Affiliations:** a Department of Neurosurgery; b Department of Ophthalmology, First Affiliated Hospital of Jiamusi University, Jiamusi, China.

**Keywords:** aneurysmal subarachnoid hemorrhage, efficacy, fasudil, safety

## Abstract

**Background:**

This study aims to systematically assess the efficacy and safety of fasudil for the treatment of aneurysmal subarachnoid hemorrhage (ASH).

**Methods:**

This study will include all of randomized controlled trials on the efficacy and safety of fasudil for the treatment of ASH. Ten electronic databases of PubMed, Embase, Cochrane Library, Google Scholar, Web of Science, Ovid, Cumulative Index to Nursing and Allied Health Literature, Allied and Complementary Medicine Database, Chinese Biomedical Literature Database, and China National Knowledge Infrastructure will be searched from inception to the May 1, 2019 without language restrictions. We will also search gray literatures to avoid missing any other potential studies. Two authors will independently perform study selection, data extraction and management, and methodologic quality assessment. The primary outcome is limbs function. The secondary outcomes comprise of muscle strength, muscle tone, quality of life, and adverse events.

**Results:**

This study will provide a comprehensive literature search on the current evidence of fasudil for the treatment of ASH from primary and secondary outcomes.

**Conclusion:**

The results of this study will present evidence to determine whether fasudil is an effective and safety treatment for patients with ASH.

**Systematic review registration:**

PROSPERO CRD42019136215.

## 1 Introduction

Aneurysmal subarachnoid hemorrhage (ASH) is a common and serious disorder that can affect brain with a variety of neurologic conditions.^[1,2]^ It is often associated with high mortality, morbidity, and also heavy healthcare burden.^[3,4]^ It has been reported that ASH accounts for 5% of all strokes; however, its mortality is about 50% (32–67%). Of these, only 30% patients can recover sufficiently to return to independent living.^[5–7]^ It is estimated that the estimated of ASH is about 10.5 per 100,000 persons each year.^[8]^ Its clinical outcomes depend on a variety of factors, such as acute bleed severity, rebleeding, as well as the presence or absence of delayed cerebral ischemia.^[9,10]^

A variety of managements can help patients with ASH, including statin, clazosentan, cilostazol, simvastatin, diltiazem, simvastatin, methylprednisolone, rehabilitation, and fasudil,^[11–23]^ especially for fasudil. Although several studies have reported that fasudil can be utilized for ASH treatment effectively,^[20–23]^ its conclusion is still unconfirmed. However, no study has systematically assessed the efficacy and safety of fasudil for the treatment of patients with ASH. Therefore, this study will explore the efficacy and safety of fasudil for ASH systematically.

## 2 Methods and analysis

### 2.1 Study registration

This study has been registered on PROSPERO (CRD42019136215). This study will follow the guidelines of Preferred Reporting Items for Systematic Reviews and Meta-analyses (PRISMA) Protocol Statement.

### 2.2 Inclusion criteria for study selection

#### 2.2.1 Participants/population

Patients diagnosed as having ASH will be included regardless their race, gender, and age.

#### 2.2.2 Interventions/exposure

Studies that assessed the efficacy and safety of fasudil on patients with ASH will be included.

The control interventions will include any other treatments except fasudil.

#### 2.2.3 Study types

We will only consider randomized controlled trials (RCTs) of fasudil for ASH. We will exclude nonclinical studies, non-RCTs, and quasi-RCTs.

#### 2.2.4 Outcome measurements

The primary outcome is limbs function, which can be any relevant scales, such as Fugl Meyer Assessment scale. The secondary outcomes comprise of muscle strength, as measured by motricity index or other related score tools; muscle tone, as assessed by modified Ashworth scale, or other relevant scales; and quality of life, as expressed by activities of daily living scale or any other specific scales. Additionally, we will also assess adverse events.

### 2.3 Search methods for the identification of studies

Ten electronic databases of PubMed, Embase, Cochrane Library, Google Scholar, Web of Science, Ovid, Cumulative Index to Nursing and Allied Health Literature, Allied and Complementary Medicine Database, Chinese Biomedical Literature Database, and China National Knowledge Infrastructure will be searched from inception to the May 1, 2019 for assessing efficacy and safety of fasudil for ASH. The search will be performed without any language restrictions. The fully reproducible search strategy is presented in Table 1 for PubMed. The similar strategy will be adapted to the other electronic databases. Additionally, we will also search gray literatures, such as dissertations, clinical trials registry, and bibliographies.

**Table 1:**
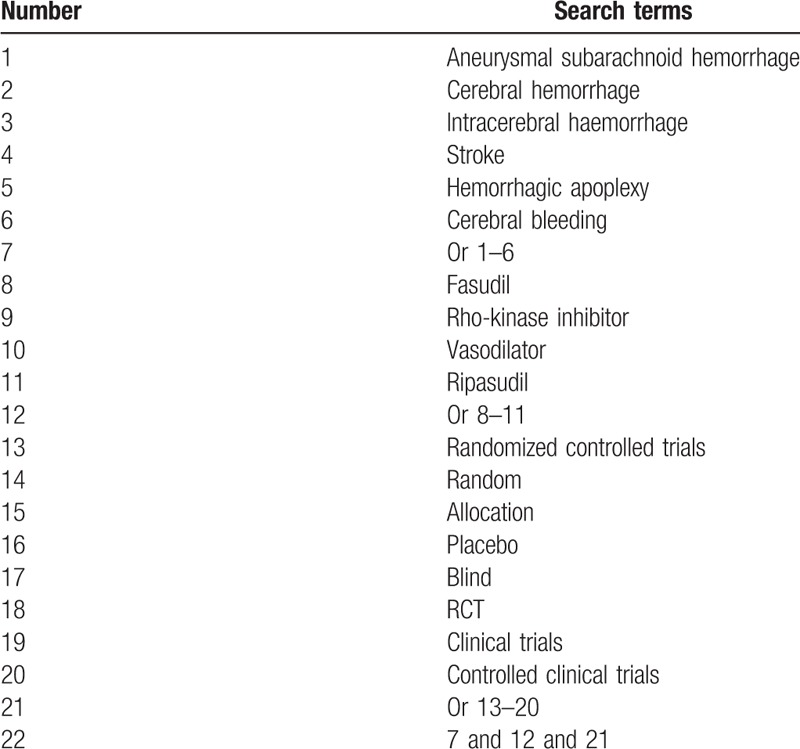
Detailed search strategy for PubMed.

### 2.4 Study selection

Two authors will independently scrutinize the literatures involved in fasudil for the treatment of ASH. We will scan the titles and abstracts 1st, and all irrelevant articles will be excluded. Then, full-texts will be read to further determine if those literatures can meet final inclusion criteria. If there are some disagreements occurred between 2 authors, a 3rd author will involve solving those disagreements through discussion. The whole procedure of study selection will be presented in the PRISMA flowchart.

### 2.5 Data extraction and management

EndNote X7 software will be utilized to manage all the records. All essential data will be extracted from eligible studies by 2 authors independently. A 3rd author will help to solve all the divergences between 2 authors regarding the data extraction. The following information will be extracted according to the previously designed form: title, author, year of publication, journal, location, patient characteristics, inclusion and exclusion criteria, study design, study methods, treatment details, and outcomes.

### 2.6 Dealing with missing data

If any essential information or data are missing or insufficient, we will contact the primary author to request that information. If we cannot receive it, then we will only analyze available data, and will discuss its potential affects.

### 2.7 Risk of bias assessment

Two authors will independently assess the risk of bias for each eligible study by using Cochrane Risk of Bias Tool. A 3rd author will involve resolving any disagreements regarding the risk of bias assessment between 2 authors. It comprises of 7 items, and each item will be further judged as 3 aspects: high, unclear, or low risk of bias for each study.

### 2.8 Reporting bias

When more than 10 eligible RCTs are entered, the Funnel plot and Egger regression test will be adopted to check if there is any reporting bias.

### 2.9 Statistical analysis

We will use RevMan 5.3 software to perform statistical analysis. We will express all continuous data as mean difference or standardized mean difference with 95% confidence intervals, and all dichotomous data as risk ratio with 95% confidence intervals.

Heterogeneity will be examined by *I*^2^ test. *I*^2^ ≤ 50% indicates reasonable heterogeneity, and a fixed-effect model will be used. *I*^2^ > 50% means significant heterogeneity, and a random-effect model will be applied. At the same time, subgroup analysis will be performed to explore the potential causes of significant heterogeneity. If it is possible, we will conduct data synthesis and meta-analysis. If it is not possible for meta-analysis performance, because of the significant heterogeneity after subgroup analysis, narrative synthesis will be reported.

Subgroup analysis will be conducted according to the different types of treatments, controls, and outcome measurements. In addition, we will also perform sensitivity analysis to check robustness and stability of pooled results by removing studies with high risk bias.

## 3 Discussion

Previous studies have reported that fasudil can be used to treat ASH effectively. However, its conclusion is still unclear, and no study has systematically addressed the efficacy and safety of fasudil for treating ASH. Therefore, this study will summarize most recent evidence to investigate the efficacy and safety of fasudil for the treatment of patients with ASH. The findings of this study will provide present evidence of fasudil for patients with ASH. In addition, its results may also provide helpful evidence for both clinical practice and patients.

## Author contributions

**Conceptualization:** Hao-yan Wang, Hong-wei Yang, Xue-feng Chang, Ren-bo Shen, Fu-yi Yang.

**Data curation:** Hao-yan Wang, Guang-fu Song, Fu-yi Yang.

**Formal analysis:** Hao-yan Wang, Guang-fu Song, Hong-wei Yang, Xue-feng Chang, Fu-yi Yang.

**Funding acquisition:** Fu-yi Yang.

**Investigation:** Fu-yi Yang.

**Methodology:** Hao-yan Wang, Guang-fu Song, Hong-wei Yang, Xue-feng Chang, Ren-bo Shen.

**Project administration:** Fu-yi Yang.

**Resources:** Hao-yan Wang, Guang-fu Song, Hong-wei Yang, Xue-feng Chang, Ren-bo Shen.

**Software:** Hao-yan Wang, Guang-fu Song, Hong-wei Yang, Xue-feng Chang, Ren-bo Shen.

**Supervision:** Fu-yi Yang.

**Validation:** Hao-yan Wang, Guang-fu Song, Hong-wei Yang, Xue-feng Chang.

**Visualization:** Hao-yan Wang, Guang-fu Song, Hong-wei Yang, Ren-bo Shen.

**Writing – original draft:** Hao-yan Wang, Guang-fu Song, Hong-wei Yang, Xue-feng Chang, Ren-bo Shen, Fu-yi Yang.

**Writing – review & editing:** Hao-yan Wang, Guang-fu Song, Hong-wei Yang, Xue-feng Chang, Fu-yi Yang.
